# Intracranial Stenting after Failure of Thrombectomy with the emboTrap^®^ Device

**DOI:** 10.1007/s00062-018-0697-x

**Published:** 2018-05-29

**Authors:** Sandra A. Cornelissen, Tommy Andersson, Ake Holmberg, Patrick A. Brouwer, Michael Söderman, Pervinder Bhogal, Leonard L. L. Yeo

**Affiliations:** 1 University hospitals Leuven, Leuven, Belgium; 2 Department of Clinical Neuroscience, Karolinska Institutet and Department of Neuroradiology, Karolinska University Hospital, Solnavägen 1, 171 77 Stockholm, Solna Sweden; 3 Department of Medical Imaging, AZ Groeninge, 8500 Kortrijk, Belgium; 4 Neuroradiology Clinic, Klinikum Stuttgart, Kriegsbergstraße 60, 70174 Stuttgart, Germany; 5 Division of Neurology, Department of Medicine, National University Health System, Singapore, Singapore

**Keywords:** Stroke, Stents, Treatment failure, Thrombectomy

## Abstract

**Background:**

Approved alternatives in the guidelines for acute ischemic stroke patients who have failed intracranial thrombectomy are lacking. Primary permanent intracranial stenting was initially used in the era before thrombectomy and might still be a useful rescue treatment in acute stroke patients suffering from ongoing large vessel occlusion refractory to thrombectomy.

**Methods:**

The prospectively collected registry of patients with acute stroke caused by large vessel occlusions and treated with the emboTrap® device in Karolinska Hospital from October 2013 through March 2017 were retrospectively reviewed. Clinical outcome of non-recanalized patients with a thrombolysis in cerebral infarction (TICI) score of 0–1 after failed thrombectomy were compared with those who were treated with permanent intracranial stenting as rescue therapy. Favorable outcome was defined as modified Rankin scale 0–2.

**Results:**

The emboTrap® device was used in 201 patients. Persistent re-occlusions on withdrawal of the thrombectomy device were seen in 26 patients (13%) and of those, 12 individuals (46%) were treated with intracranial stenting. Baseline National Institutes of Health stroke scale (NIHSS), occlusion site, and onset-to-puncture time did not differ between the stenting group and the non-recanalized group. During the procedure half dose (5/12 patients) or full dose abciximab (6/12 patients), or aspirin (1/12 patient) was given intravenously immediately after stent placement. In 2 patients (17%) multiple stents were implanted. The stenting group had better functional outcomes at 3 months compared to the non-stenting group with 8/12 (66%) vs. 3/14 (21.4%, *p* < 0.05). Of the patients 5 (36%) in the non-stented group had died at 3 months follow-up, whereas mortality in the stenting cohort was 0% (*p* < 0.05) and no symptomatic intracranial hemorrhage (ICH) occurred in either group.

**Conclusion:**

Intracranial stenting after failure of recanalization with thrombectomy led to a better rate of clinical outcome than leaving the patient non-recanalized. The required antiplatelet therapy, predominantly abciximab, did not lead to additional ICH.

**Electronic supplementary material:**

The online version of this article (10.1007/s00062-018-0697-x) contains supplementary material, which is available to authorized users.

## Introduction

Acute ischemic stroke (AIS) is still the number one cause of dependency in the world, but there has been a large change in our ability to treat cerebral large vessel occlusions (LVO). Thrombectomy is a now a first-line therapy for the management of AIS occurring in the anterior circulation due to LVO [1–4]. Newer generations of thrombectomy devices have been designed to improve the rates of recanalization and reduce complications; however, in 29% of AIS patients treated with mechanical thrombectomy (MT) recanalization is not successful [5]. Recanalization of occluded intracranial arteries via stenting was initially used as an experimental treatment in AIS [6–11] but was quickly supplanted by the newer superior thrombectomy techniques. Nonetheless, these early trials suggested that stenting in AIS with LVO could be a plausible method to restore the patency of vessels which re-occlude after thrombectomy.

The antiplatelet therapy after intracranial stenting is still controversial and lacking guidelines as to the optimal treatment regimen. The situation is more complicated when an intracranial stent is implanted in the setting of an acute stroke where there is no time to pre-load the patient and test the platelet activity to tailor the medical treatment. There is also a higher potential risk of intracranial bleeding due to hemorrhagic conversion in the infarcted brain tissue and procedural complications [12].

This study included a cohort of AIS patients in whom recanalization failed after MT with the emboTrap® (Cerenovus, Johnson and Johnson, New Brunswick, NJ, USA) second generation thrombectomy device. The study aimed to determine if the deployment of an intracranial stent was an effective strategy in such resistant cases.

## Methods

The prospectively maintained registry of patients with AIS due to LVO who were treated with the emboTrap^®^ device in our center between October 2013 and March 2017 was retrospectively reviewed. The clinical outcome of non-recanalized patients with a thrombolysis in cerebral infarction (TICI) score 0–1 after failed thrombectomy was compared with non-recanalized patients who were treated with permanent intracranial stenting as rescue therapy after failed thrombectomy.

The standard approach in thrombectomy procedures is described in the supplementary file. In the non-recanalized patients it was an surgeon’s decision to either leave the vessel non-recanalized or to perform rescue stenting. There was no pre-determined number of failed attempts or mandatory trial of intra-arterial nimodipine or glyceryl trinitrate (GTN). In several of these patients, aspiration thrombectomy as rescue therapy was attempted through the intermediate catheter but was unsuccessful. Because there are no guidelines available on how to proceed in such patients, the decision to perform stenting after failed thrombectomy was completely dependent on the surgeon.

When it was decided to perform stenting in a non-recanalized patient an angiographic run was first performed through either the microcatheter or the balloon guide catheter (BGC)/intermediate catheter to measure the stenosis and to choose the size of the stent to be used. No balloon pre-dilatation is usually done. The choice of intracranial stent was left to the operator’s preference, although in our center either the Enterprise® stent (Codman Neurovascular, Raynham, MA, USA) or the Solitaire™ AB stent (ev3 Inc., Plymouth, MN, USA) tended to be used. Sizing of the vessel was based on standard techniques.

In conjunction with deployment of the intracranial stent half or full bolus dose of weight-adapted abciximab (Eli Lilly, Indianapolis, IN, USA) or aspirin was given intravenously. An intravenous abciximab infusion was not routinely used. Post-deployment angioplasty of the implanted stent was left to the operator’s discretion and was typically performed in cases of persistent flow restriction after deployment of the intracranial stent.

A dual-energy computed tomography (CT) scan was performed 22–36 h after the procedure. If there were no contraindications on the imaging studies, dual antiplatelet therapy of aspirin in combination with either clopidogrel or prasugrel was commenced (Table 2). This was continued for 3–6 months and subsequently the patients were de-escalated to 100 mg aspirin for life. Antiplatelet activity was checked using the Multiplate test (Verum Diagnostica, Munich, Germany) within 3 days after the procedure and if there was evidence of sub-optimal inhibition the anti-platelet medication was adjusted.

Details of the patients’ demographics, National Institutes of Health stroke scale (NIHSS) score at presentation, the procedural duration and the intervals from symptom onset to imaging, groin puncture, and reperfusion (or end of procedure in non-recanalized patients in whom no stenting was performed) were collected. The pre-images and post-images of each thrombectomy attempt, and the final post-procedural images were core laboratory assessed for modified thrombolysis in cerebral infarction (mTICI) score after each retrieval attempt [13]. Successful recanalization was defined as mTICI ≥ 2b, independently assessed for each patient by the external core laboratory physician (AM). Favorable functional outcome was defined as ≤2 on the modified Rankin Score (mRS) at 3 months and assessed by a neurologist not involved in the acute care. Any hyperdensity seen on the 24 h dual energy CT control images, even if due to contrast media, was scored as a hemorrhagic occurrence by the core laboratory. Symptomatic intracranial hemorrhage was defined as any hyperdensity with a worsening of the NIHSS score by ≥4 points [14].

## Statistical Methods

The numerical variables are presented as mean and standard deviation or median and range. Categorical variables are presented as percentages. Numerical predictors were tested by using 2‑sample *t-*test or Mann-Whitney *U* test where applicable. Categorical variables were evaluated using χ^2^-test or Fisher’s exact test where applicable. Only univariate analysis was performed in view of the small number of patients, which precluded multivariate analysis. Statistical analyses were performed using the Statistical Package for Social Sciences (SPSS) version 21 (IBM Corp., Armonk, NY, USA). Institutional ethics review board approval for this study was obtained from the regional ethical committee in Stockholm (regionala etikprövningsnämnden i Stockholm) Diarienr:2016/1041-31/4.

## Results

In this study 201 consecutive cases of AIS treated with the emboTrap® device were reviewed. Of the patients 26 showed persistent re-occlusion after withdrawal of the thrombectomy device and of those, 12 individuals were treated with implanted stents and 14 were left non-recanalized. A single Enterprise® stent was used in 6 patients, and 2 telescoping Enterprise® stents were implanted in 2 of the patients. The Solitaire AB stent was inserted in 4 patients.

Baseline characteristics, stroke severity, occlusion site, number of thrombectomy attempts, onset-to-puncture time, and puncture-to-reperfusion time did not differ significantly between the stented and non-stented groups (Table 1). After stenting, in one patient the Solitaire stent (1/12, 8%) occluded immediately after deployment. In 4/12 (33%) patients a flow-limiting stenosis was present after stent placement, which was treated by balloon angioplasty in 4 patients (1 with a Scepter balloon, Microvention, Saint-Germain-en-Laye, France, 3 with a Gateway™ balloon, Boston Scientific, Fremont, CA, USA).

**Table 1:** Comparison of features of the non-recanalized group vs. stented group

	Non-recanalized (*n* = 14)	Intracranial stenting (*n* = 12)	*P*-value
Age, mean (SD)	67.3 years (9.5)	65.2 years (13.9)	0.647
Male	11 (78.6%)	6 (50%)	0.133
Recent stroke	1 (7.1%)	0 (0%)	0.538
NIHSS, median (range)	16.5 (8–22)	16.5 (5–22)	0.432
Hypertension	4 (28.6%)	3 (25%)	0.838
Atrial fibrillation	3 (21.4%)	2 (16.7%)	0.759
IV tPA	6 (42.9%)	3 (25%)	0.296
Premorbid mRS, median (range)	0 (0–3)	0 (0–1)	0.166
Onset-to-puncture time, mean (SD)	270 mins (176.5)	288 mins (150.3)	0.626
Puncture-to-recanalization time, mean (SD)^a^	83.5 mins (50.6)	83.3 mins (50.1)	0.985
mRS, median (range)	4 (1–6)	2 (1–5)	*0.007*
mRS 0–2	3 (21.4%)	8 (66.0%)	*0.026*
Mortality	5 (35.7%)	0 (0%)	*0.030*
Pre-treatment mTICI, (range)	0 (0–1)	0 (0–1)	–
mTICI final	0. 7 (50%) 1. 7 (50%)	2a–1 (8.3%) 2b–9 (75%) 3–2 (16.7%)	*<0.001*
Any bleeding	4 (28.6%)	0 (0%)	0.067
sICH	0 (0%)	0 (0%)	0.958
Occlusion location	Basilar 4 (28.6%) M1 9 (64.3) M2 1 (7.1%)	Basilar 2 (16.7%) Vert 1 (8.3%) TICA 1 (16.7%) M1 8 (66%)	0.453
Number of attempts, median (range)	4 (1–7)	3.5 (1–8)	0.952
Infarct size, ml (SD)	30.1 (36)	14.9 (22.3)	0.227

*SD* standard deviation *NIHSS* National Institutes of Health stroke scale, *IV tPA* intravenous tissue plasminogen activator, *mRS* modified Rankin scale, *mTICI* modified thrombolyisis in cereberal ischemia scale, *sICH* symptomatic intracranial hemorrhage, *Vert* vertebral artery, *TICA* terminal internal cartoid artery, *M1* M1 portion of middle cerebral artery, *M2* M2 portion of middle cerebral artery, *Basilar* basilar artery

^a^in the non-recanalized patients instead of puncture-to-recanalization time, the time of puncture-to-end-of-procedure is given

The stenting group had a significantly better rate of mRS 0–2 (66% versus 21%) than the non-stenting group (Table 1). None of the stented patients died, while 5 patients in the non-stented group had died at 3 months follow-up (*p* < 0.05). Out of 12 patients 11 (92%) showed mTICI 2b or better reperfusion at the end of the procedure after intracranial stenting, patient number 8 had persisting occlusion despite stenting. All of the 14 patients in the non-stenting group exhibited mTICI 0–1, 4 out of 14 individuals in the non-stenting group (29%) had core laboratory-assessed high-density lesions on follow-up CT but none of these patients had symptomatic intracranial hemorrhage (ICH) (Table 1). None of the stented patients had radiological evidence of ICH despite treatment with full dose abciximab in 6 patients and half-dose abciximab in 5 patients. Of these stented patients three had been treated with intravenous tissue plasminogen activator (IV tPA) before the thrombectomy procedure and o patient received intravenous aspirin before stenting rather than abciximab. (Table 2). In 75% of the cases a stenosis of the stent was present in the final angiographic run after deployment.

**Table 2:** Details and outcomes of the 12 patients who underwent intracranial stenting

S/*N*	1	2	3	4	5	6	7	8	9	10	11	12
Type of stent	Enterprise® stents, 4 × 16 and 4 × 23	Enterprise® 4 × 39	Enterprise® stents, 4 × 39 and 4 × 16	Enterprise® 4 × 30	Solitaire™ 4 × 15	Enterprise® 4 × 28	Enterprise® 4 × 20	Solitaire™ 4 × 20	Enterprise® 4 × 23	Solitaire™ 6 × 20	Enterprise® 4 × 23	Solitaire™ 4 × 20
Location of stent placement	Right M1	Basilar	Right vertebral	Left M1	Left M2	Left M1	Left vertebral	Right M1	Right M1	Left M1	Left M1	Right M1
State of vessel immediately postdeployment	Stenosis	Tight stenosis (flow limiting)	Stenosis	Near occlusion (flow limiting)	Near occlusion (flow limiting)	Patent	Stenosis	Occluded	Tight stenosis (flow limiting)	Tight stenosis	Stenosis	Tight stenosis
PTA post deployment	No	Scepter balloon	No	Gateway balloon	Gateway balloon	No	No	No	Gateway Balloon	No	No	no
State of vessel on repeat serial imaging	No repeat vessel scan	Patent on day 1 CTA + 6 mth DSA	Patent on day 1 MRA and day 4 CTA	Stenosis on day 1 CTA but patent on 6 mth CTA	Patent on day 1 CTA but Occlusion on 6 mth CTA	Patent on day 1 and 12 mth CTA	Stenosis on day 3 MRA. patent on 8 mth CTA	No repeat vessel scan	Patent on 1 mth CTA	Irregular lumen on 6 mth CTA. Patent on 12 mth and 24 mth CTA	Patent on 6 mth CTA	Patent on 1 year CTA
Pre-stenting medication	Full dose abciximab	Full dose abciximab	Full dose abciximab	Full dose abciximab	Half dose abciximab	Full dose	Full dose abciximab	Half dose abciximab	Half dose abciximab	IV Aspirin	Half dose abciximab	Half dose abciximab
mTICI at end of procedure	3	2b	2b	2b	2b	3	2b	2a	2b	2b	2b	2b
mRS at 3 months	2	1	4	2	3	1	2	5	1	2	2	3
Postprocedural antiplatelet regimen	aspirin 75 mg and clopidogrel 75 mg	aspirin 75 mg and clopidogrel 75 mg	aspirin 75 mg and prasugrel 10 mg for 6 mths	aspirin 75 mg and prasugrel 10 mg for 6 mths	aspirin 75 mg with LMWH for lung emboli	aspirin 75 mg and prasugrel 10 mg for 3 mths	aspirin 75 mg and prasugrel 10 mg for 6 mths	aspirin 75 mg	clopidogrel 75 mg and LMWH	clopidogrel 75 mg and warfarin for 3 mths for mechanical heart valves	aspirin 75 mg and prasugrel 5 mg for 6 mths	aspirin 75 mg and prasugrel 10 mg for 3 mths

*MRA* magnetic resonance angiography, *CTA* computed tomography angiography, *DSA* digital subtraction angiography, *mTICI* modified thrombolysis in cerebral infarction scale, *mRS* modified Rankin scale, *LMWH* low molecular weight heparin, *ICH* intracranial hemorrhage, *PTA* percutaneous transluminal angioplasty, *mth* months

Imaging follow-up, whether computed tomography angiography (CTA), magnetic resonance angiography (MR-A) or angiography, was performed in 10/12 stented patients. The mean follow-up duration was 8 months (range 1–24 months), 9 (90%) stents were patent on imaging follow-up, no imaging follow-up was performed on 1 stent and 2 stents were occluded (1 immediately after placement, the other stent was patent at day 1, but occluded on CTA after 6 months). Of the 10 patients with repeat vascular imaging 6 (60%) had a stenosis after stent deployment but went on to have good functional outcomes at 3 months despite the stenosis (Table 2).

## Discussion

This series adds data to validate the fact that intracranial stenting might be a viable alternative in cases where a second-generation stent retriever-based MT has failed. It also shows that intravenous abciximab is a reasonably safe and effective drug for anti-platelet aggregation to use in an acute stroke setting with potentially little risk of hemorrhage. In three patients, abciximab was used after treatment with IV tPA, which did not lead to hemorrhage in these patients.

Recanalization rates for intracranial LVO are >80% with modern devices; however, a significant subset of patients remains in whom placement of the stent-retriever leads to temporary recanalization, but withdrawal results in re-occlusion. This might be due to an underlying atherosclerotic stenosis or a fibrinous thrombus, which is too adherent to the vessel wall and allows the stent to be withdrawn through it without gathering up the clot. With each additional thrombectomy attempt, there are concerns for vessel wall injury, thrombus fragmentation with embolization and ongoing damage of the ischemic brain [15, 16].

The Enterprise® stent and Solitaire™ AB are self-expanding closed-cell stents, which were initially designed to assist in the coiling of brain aneurysms. They have been employed as a revascularization device for use in AIS after other devices have failed [5, 17]. While the closed cell design provides good radial force, a study using the Enterprise® for primary stenting during AIS suggested that balloon angioplasty after stent deployment was required in 45% of the cases [10]. This series contradicts this finding with 8 out of 10 patients having stenosis post-deployment that resolved on its own as seen on subsequent imaging. Only 4 of these patients underwent balloon angioplasty after stent deployment when the stenosis appeared to be flow limiting, while the rest relied on the radial force of the stent to gradually open the stenosis. In the last 2 patients with near occlusive stenosis after stenting, no angioplasty was done, and the follow-up scans showed patency of the stented portion.

To our knowledge there is only a single study, which showed better outcomes in patients who underwent permanent stenting after failed thrombectomy compared to patients who did not [5]. Our study validated these findings and while permanent stenting should still only be a final recourse, it reassures us that it is a valid alternative when thrombectomy has failed.

There is a risk of in-stent reocclusion or stenosis after intracranial stenting and the antiplatelet regimen to prevent this is still controversial [18–22].  A recent publication suggested that glycoprotein IIb/IIIa inhibitor during rescue stenting for failed thrombectomy was associated with less in-stent restenosis and better 3‑month mRS scores [23]. Of the stented patients in this study 92% were treated with abciximab during stent implantation and there were no radiological signs of ICH in the stented cohort compared to 4 patients in the non-stented cohort who developed ICH. The low rate of symptomatic ICH and high rate or mTICI 2b/3 recanalization (83.3%) in the stented group is similar to the results achieved by previous studies in which stenting was performed during acute stroke (Table 3).

**Table 3:** Results of intracranial stenting in stroke patients reported in the literature

	*N*	Devices	Location	Median NIHSS	mRS 0–2	sICH	Reperfusion
*Pre-stent retriever era*							
Levy et al. [6]	19	Neuroform® and Wingspan	8 M1, 1 M2, 3 VB, 4 TICA, 3 ICA	18 (2–23)	4/18	2/18	15/19 TIMI 2/3
Zaidat et al. [7]	9	Neuroform® and Wingspan	5 M1, 1 M3, 2 ICA, 1 VB	18	6/9	1/9	8/9 TIMI 2/3
Brekenfeld et al. [8]	12	Wingspan	5 M1, 1 ACA, 6 VB	14 (5–38)	3/12	0/12	11/12 TIMI 2/3
Sung et al. [9]	10	Wingspan and Gateway™ balloon	10 M2	13.8 (6–23)	6/10	0/10	9/10 TICI2b/3
Dumont et al. [10]	20	Enterprise® stent	2 BA, 14 MCA, 4 ICA	15.5 (8–25)	10/20	1/20	18/20 TIMI2/3
Kulcsár et al. [11]	6	Enterprise® stent	2 MCA, 4 TICA	14 (9–17)	1/6	2/6	4/6 TICI 2b/3
Levy, et al. [22]	20	Wingspan stent	16 MCA, 3 BA, 1 TICA	13	9/20 mrs 0–1 at 1 mth	1/20	20 TIMI2/3
*Post-stent retriever era*							
Baracchini et al. [17]	23	Solitaire™ AB	2 ICA, 18 MCA, 3 TICA	16 (4–26)	13/23	1/23	17/23 mTICI 2b/3
Baek et al. [5]	17	Solitaire™ AB	7 ICA, 10 MCA	19 (7–22)	6/17	2/17	14/17 mTICI 2b/3
Delgado Acosta et al. [24]	42	Enterprise® and Gateway™ balloon	13 BA, 14 TICA, 15 MCA	Carotid 17, basilar 26	16/42	2/42	30/42 mTICI 2b/3
Woo et al. [25]	27	13 Solitaire™ FR, 14 others	10 ICA,13 MCA, 4 BA	16 (8.5–20.5)	8/27	3/24	22/27 mTICI 2b/3

*M1* M1 portion of middle cerebral artery, *M2* M2 portion of middle cerebral artery, *VB* vertebrobasilar, *TICA* terminal internal carotid artery, *ICA* internal carotid artery, *TIMI* thrombolysis in myocardial infarction, *TICI* thrombolysis in cerebral infarction

Several limitations of our study should be acknowledged. The small sample size of 12 patients may not be representative and a larger series should be carried out to validate the study results. This study reviewed patients treated with the emboTrap® device and therefore it is uncertain as to whether the results can be extrapolated to MT performed with other thrombectomy devices. Nevertheless, other studies showed similar outcomes and reperfusion rates. (Table 3) Only 10 out of 12 patients had repeat vascular imaging and not all of them had MRI after the procedure. Another major limitation is that we were unable to ascertain the underlying cause of reocclusion post-thrombectomy and it could be due to a fibrinous clot, a dissection or vasospasm. A high-resolution vessel MRI could be useful to determine the cause; however, the limited time in an acute stroke setting precludes such investigations from being done. Finally, the lack of a standardized approach for both treatment and antiplatelet regimen choices is a limitation, which resulted in inhomogeneous treatment cohorts and a selection bias cannot be ruled out. Moreover, the patients did not switch to direct aspiration, which would be a reasonable alternative before permanent stenting.

## Conclusion

Permanent intracranial stenting might be a possible salvage modality in patients suffering from failed thrombectomy procedure with promising rates of favorable clinical outcome. The use of abciximab appears to be reasonably safe in the setting of acute intracranial stenting for failed MT. Complete restoration of vessel caliber does not appear to be necessary to achieve a good clinical outcome. These findings should be validated in a larger series.

## Caption Electronic Supplementary Material

The protocol followed by Karolinksa hospital for stroke thrombectomy.
