# Supplementary material for: Intracranial Stenting after Failure of Thrombectomy with the emboTrap® Device
Source: Clin Neuroradiol. 2018 May 29;29(4):677–83. doi: 10.1007/s00062-018-0697-x (PMC6884426; doi:10.1007/s00062-018-0697-x)
Supplement: Supplementary file 1 — The protocol followed by Karolinksa hospital for stroke thrombectomy. [file 62_2018_697_MOESM1_ESM.docx]

**Supplemental file:**

**Description of the thrombectomy method usually performed:** We perform mechanical thrombectomy in our center for large vessel occlusion strokes regardless of time of onset if the physician decides that there is adequate salvageable tissue. Patients arriving within the 4.5-hour time-window receive intravenous thrombolytic treatment if there is no contraindication. We perform CT and CT-perfusion upon arrival and the vasculature is reconstructed from the CT-perfusion images. Each interventionalist has the freedom to decide on the device and technique. Commonly, in the anterior circulation, an 8 or 9 French balloon guide catheter (BGC) is advanced to the cervical internal carotid artery with the support of a long 90cm Arrow sheath (Teleflex, USA). Whereas in the posterior circulation a 6 French Envoy guiding catheter is advanced through a 60cm arrow sheath into either vertebral artery. A 0.027 microcatheter is navigated through the occlusion and the EmboTrap (Neuravi/Cerenovus) is optimally positioned into the clot (figure 1). After inflation of the balloon, the EmboTrap is retracted into the BGC with manual aspiration on the guiding catheter using a 50cc syringe for gentle flow reversal. Alternatively, a distal access catheter is sometimes used and positioned just proximal to the occlusion, and the EmboTrap is fully or partially retrieved into it under aspiration in both the distal access catheter and the BGC. Standard angiographic runs are taken before and after the procedure and between thrombectomy attempts based on operator preference.
